# THE ASSOCIATION BETWEEN RELIGIOSITY AND COGNITIVE FUNCTIONING AMONG OLDER ADULTS WITH HEMATOLOGICAL CANCERS

**DOI:** 10.1093/geroni/igac059.937

**Published:** 2022-12-20

**Authors:** Jessica Krok-Schoen, Ashley Rosko, Ying Huang, Allesia Funderburg, Katy Dvorak, Erin Stevens

**Affiliations:** The Ohio State University, Columbus, Ohio, United States; The Ohio State University Comprehensive Cancer Center, Columbus, Ohio, United States; The Ohio State University, Division of Hematology, Columbus, Ohio, United States; The Ohio State University Comprehensive Cancer Center, Columbus, Ohio, United States; The Ohio State University Comprehensive Cancer Center, Columbus, Ohio, United States; The Ohio State University Comprehensive Cancer Center, Columbus, Ohio, United States

## Abstract

Religiosity is positively associated physical health outcomes, yet longitudinal research on this association among older adults with cancer is limited. Participants were enrolled in a longitudinal study assessing chemotherapy’s impact on the health of older adults with hematological cancers. Religiosity was assessed by the Duke University Religion Index (DUREL), with higher scores indicating higher religiosity. Cognitive functioning was assessed by the Blessed Dementia Scale, with higher scores indicating more impairment. Participants (n=97) had a median age of 70 years (range:60-88) and were 58% male and 97% white. DUREL scores were unchanged with a mean score of 22 (range:5-27). Cognitive functioning significantly improved with a median score of 4 (range:0-22) at baseline to 0 (range:0-20) at end-of-treatment. Religiosity was not significantly correlated with cognitive functioning over time. Future research should explore the trajectory of cognitive functioning and other sources of coping (social support, palliative/supportive care) among older adults with hematological cancers.

